# Untargeted metabolomic analysis of dietary rumen-protected choline supplementation in fattening lambs

**DOI:** 10.3389/fvets.2025.1666044

**Published:** 2025-10-24

**Authors:** Jinyan Yun, Airong Zhu, Peihua You, Xuezhao Sun

**Affiliations:** ^1^College of Animal Science and Technology, Jilin Agricultural Science and Technology College, Jilin City, Jilin Province, China; ^2^Jiangsu AgriPortal Feed Co., Ltd., Nanjing City, Jiangsu Province, China; ^3^Jilin Inter-regional Cooperation Centre for the Scientific and Technological Innovation of Ruminant Precision Nutrition and Smart and Ecological Farming, Jilin City, Jilin Province, China; ^4^Bioeconomy Science Institute, Grasslands Research Centre, Palmerston North, New Zealand

**Keywords:** untargeted metabolomics, rumen-protected choline, fattening lambs, serum metabolites, lipid metabolism, amino acid metabolism, LC–MS/MS, KEGG pathway analysis

## Abstract

**Introduction:**

Choline is an essential nutrient that plays a key role in lipid metabolism and growth performance in livestock.

**Methods:**

This study investigated the effects of rumen-protected choline (RPC) supplementation on serum metabolite profiles in fattening lambs. Twenty 3-month-old hybrid rams (Small-Tailed Han × Northeast Fine-Wool; initial body weight: 15.3 ± 1.8 kg) were randomly assigned to two groups (*n* = 10 each): a control group (CON; 0 g RPC/kg dry matter [DM]) and an RPC group (5 g RPC/kg DM). Over a 122-day feeding period, lambs were provided *ad libitum* access to feed and water, with feedings at 08:00 and 15:00 daily. Serum samples were collected at the end of the trial and analyzed using untargeted metabolomics based on liquid chromatography–tandem mass spectrometry (LC-MS/MS).

**Results:**

A wide range of metabolites were identified, including benzenoids, lipids and lipid-like molecules, nucleosides and nucleotides, organic acids, and derivatives. Pathway analysis using the Kyoto Encyclopedia of Genes and Genomes (KEGG) revealed involvement in lipid, amino acid, vitamin, and carbohydrate metabolism. Partial least squares-discriminant analysis (PLS-DA) showed clear separation between CON and RPC groups. Triacylglycerol, L-methionine, plasmenylcholine, taurocholate, 1-acyl-sn-glycero-3-phosphoethanolamine, and 1-acyl-sn-glycero-3-phosphocholine were identified as potential bio markers associated with increased hot carcass weight (HCW) and slaughter rate.

**Discussion:**

RPC supplementation significantly modulated the serum metabolome, enhancing HCW and slaughter rate, likely via lipid, amino acid, and energy metabolism pathways.

## 1 Introduction

Choline, a quaternary ammonium compound [trimethylaminoethanol or (2-hydroxyethyl)trimethylammonium], is an essential nutrient critical for various physiological processes, including the synthesis of acetylcholine and phosphatidylcholine, which are vital for cell membrane integrity, methylation reactions, lipid transport, and neurotransmission (1). In addition, choline alleviates inflammation and oxidative stress by enhancing hepatic fatty acid utilization, thereby reducing the risk of fatty liver disease (2, 3). Dietary choline deficiency can elevate reactive oxygen species in liver mitochondria, leading to oxidative stress that impairs hepatic secretory functions and reduces circulating lipoprotein levels (4). While standard livestock diets typically contain choline, its bioavailability is often insufficient to meet physiological demands (5, 6).

Choline deficiency is particularly pronounced in ruminants. The rumen microbiota can degrade choline, significantly reducing the amount of choline available for absorption in the small intestine. This degradation process results in even more severe choline deficiency in ruminants compared to swine and poultry (7). To address this challenge, rumen-protected choline (RPC) has been developed to shield choline from ruminal degradation, ensuring its delivery to the small intestine for efficient absorption (5, 7). RPC can enhance choline availability in ruminant diets, thereby better meeting physiological needs.

RPC has become increasingly prevalent in intensive animal husbandry, particularly in dairy production (7, 8). It has been shown to improve milk production (9), reproductive outcomes (10, 11), and meat quality (12, 13), while reducing morbidity and mortality rates (14, 15). In meat-producing ruminants, RPC supplementation has demonstrated notable effects. Some studies have reported that RPC increases final body weight in fattening beef cattle (16), improves growth performance and feed utilization efficiency (17, 18), and enhances both growth and meat quality in fattening lambs (19) and goats (12, 20), resulting in significant economic benefits (11–14). However, other studies have found no significant effects of RPC on daily feed intake, feed-to-gain ratios, or growth and slaughter performance in fattening lambs (21, 22).

The mechanisms underlying the effects of RPC supplementation in ruminants remain unclear. To explore the molecular mechanisms of RPC’s influence on growth performance and lipid metabolism in fattening lambs, we propose the following hypothesis: Dietary RPC supplementation alters the serum metabolite profile in fattening lambs, thereby affecting their growth performance and lipid metabolism. To test this hypothesis, this study employs untargeted liquid chromatography–tandem mass spectrometry (LC–MS/MS) metabolomics to investigate the effects of RPC supplementation on serum metabolites in fattening lambs. By analyzing the metabolic changes induced by RPC supplementation, we aim to elucidate its mechanisms of action. Furthermore, we intend to identify differentially abundant metabolites as potential biomarkers for assessing RPC’s impact in ruminant nutrition. The findings of this study will provide valuable insights into the effects of RPC supplementation in ruminants and offer theoretical support for its practical application.

## 2 Materials and methods

### 2.1 Ethics statement

All experimental procedures involving animals were approved by the Animal Ethics and Welfare Committee of JiLin Agricultural Science and Technology College, Jilin City, Jilin Province, China (Approval No. 2019001). The study was conducted at the Animal Experimental Station of JiLin Agricultural Science and Technology College, Jilin City, Jilin Province, China.

### 2.2 Animals and experimental design

Twenty healthy 3-month-old hybrid Small-Tailed Han and Northeast Fine-Wool rams (initial body weight: 15.3 ± 1.8 kg) were transitioned from hay to a pelleted total mixed ration over 7 days (days −12 to −6), followed by a 5-day adaptation period (days −5 to −1). On day 1, lambs were randomly assigned to two groups (*n* = 10 per group): control (CON, 0 g/kg dry matter [DM] RPC) and RPC (5 g/kg DM RPC). The experiment spanned 122 days and consisted of fattening period 1 (days 1–56), fattening period 2 (days 57–112), and a digestibility measurement period (days 113–122). Blood samples for metabolomics were collected on day 122. Lambs were slaughtered at the end of the experiment. Results on growth performance, digestibility, and slaughter performance were previously reported (22).

Animals were sampled for blood collection prior to the morning feeding, following 122 days of RPC supplementation. Blood was drawn from the jugular vein into coagulation-promoting tubes with separating gel (Sanli Industrial Co., Ltd., Huizhou, China). The samples were centrifuged at 1,000 × *g* for 10 min using a TDL-80-2B centrifuge (Anting Scientific Instrument Factory, Shanghai, China). The resulting serum was then stored at −80 °C.

### 2.3 Feed and management

Rumen-protected choline containing 25% choline chloride was sourced from Shandong Fulikang Animal Nutrition Co., Ltd., Binzhou, Shandong, China. The pelleted total mixed ration was formulated according to the Chinese Fattening Feed Standard (23) and processed by the Tongliao Branch of Jiangsu AgriPortal Feed Co., Ltd. Lambs were fed twice daily at 08:00 and 15:00 with equal portions, ensuring *ad libitum* access to feed and water. Feed allowance was adjusted daily to achieve approximately 10% refusal, based on prior intake. Before the experimental period, lambs received an oral dose of albendazole (15 mg/kg body weight) for deworming. Daily records of weather conditions, temperature, humidity, and animal behavior were maintained to monitor the environment and ensure animal welfare (22). At the end of the trial, all lambs were humanely slaughtered. Electrocution was used to ensure immediate loss of consciousness, followed by exsanguination through jugular vein incision. These procedures were conducted by certified veterinary technicians under the direct supervision of a licensed veterinarian.

### 2.4 Metabolomics analysis

#### 2.4.1 Metabolite extraction

Serum samples were thawed on ice, and 20 μL of each sample was mixed with 120 μL of pre-cooled 50% methanol, vortexed for 1 min, and incubated at room temperature for 10 min. The mixture was stored at −20 °C overnight, then thawed and centrifuged at 4,000 × *g* for 20 min. Supernatants were transferred to 96-well plates and stored at −80 °C until liquid chromatography-mass spectrometry (LC–MS) analysis. Pooled quality control (QC) samples were prepared by combining 10 μL of each extraction mixture to monitor analytical stability.

#### 2.4.2 LC–MS analysis

Untargeted metabolomics analysis was performed on 25 serum samples (including 20 biological samples + 5 QC injections) using a high-performance liquid chromatography (HPLC) system coupled with a TripleTOF 5,600 + high-resolution mass spectrometer (SCIEX, United Kingdom) in both positive and negative ion modes. Chromatographic separation was conducted using an ACQUITY UPLC T3 column (100 mm × 2.1 mm, 1.8 μm; Waters, United Kingdom) maintained at 35 °C, with a flow rate of 0.4 mL/min. The mobile phase consisted of solvent A (water with 0.1% formic acid) and solvent B (acetonitrile with 0.1% formic acid). The gradient elution profile was as follows: 0–0.5 min, 5% B; 0.5–7 min, 5 to 100% B; 7–8 min, 100% B; 8–8.1 min, 100 to 5% B; 8.1–10 min, 5% B. The injection volume was 4 μL. To ensure system stability, a QC sample was analyzed after every 10 samples. Mass spectrometry data were processed using XCMS software for peak detection and metaX software for compound quantification and differential analysis. Primary and secondary mass spectra were annotated using metaX and an in-house reference database, respectively.

### 2.5 Statistical analysis

Raw mass spectrometry data were converted to mzXML format using MSConvert (ProteoWizard). Peak extraction and quality control were performed using XCMS, followed by adduct and ion annotation with CAMERA software. Metabolites were identified by matching primary and secondary mass spectrometry data against an in-house reference standard database, with annotations supported by the Human Metabolome Database (HMDB)^1^ and Kyoto Encyclopedia of Genes and Genomes (KEGG)^2^ for physicochemical and biological characterization.

Quantitative analysis and differential metabolite screening were conducted using metaX software. Differences in metabolite concentrations between groups were assessed using Student’s t-tests, with *p*-values adjusted for multiple comparisons using the false discovery rate (FDR, Benjamini-Hochberg method). Supervised partial least squares-discriminant analysis (PLS-DA) was performed using metaX to identify discriminatory variables between groups, with a variable importance in projection (VIP) threshold of 1.0 used to select significant features.

Pearson’s two-tailed correlation coefficients were calculated to assess the relationships between differentially abundant metabolites (DAMs) and production traits, including hot carcass weight (HCW) and slaughter rate. A correlation network diagram was subsequently constructed to visualize these associations.

## 3 Results

### 3.1 Metabolite identification

To investigate the metabolic effects of RPC supplementation in fattening lambs, serum samples were collected from two groups after a 122-day experimental period: a control group (CON, no RPC) and an RPC-supplemented group (5 g/kg DM). Untargeted metabolomics analysis was performed using high-resolution liquid chromatography–tandem mass spectrometry (LC–MS/MS) in positive (POS) and negative (NEG) ion modes. Raw data were converted to mzXML format using MSConvert (ProteoWizard) for peak extraction with XCMS software, and the metabolites were identified based on mass-to-charge ratio (m/z), retention time, and chromatographic peak area. Metabolites were annotated by matching primary m/z and secondary fragment ion data against an in-house reference database.

**Figure 1:**
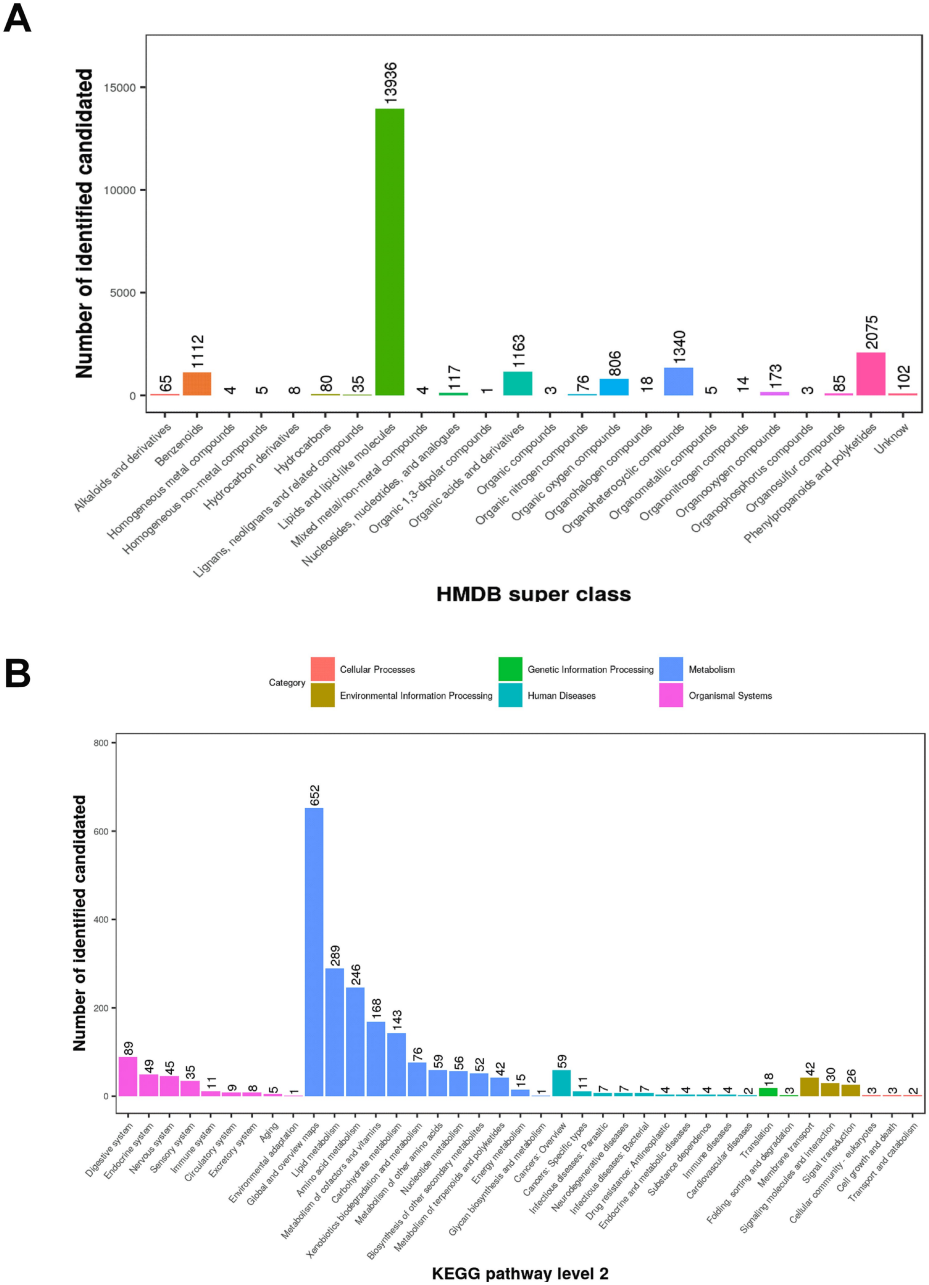
HMDB superclass categories and KEGG pathways of MS1 metabolites in positive ion mode (POS). **(A)** HMDB superclass: Metabolites identified with level-one confidence were classified into 24 HMDB superclasses. The x-axis shows the superclasses, and the y-axis represents the number of possible metabolites. **(B)** KEGG pathway (level 2): The x-axis shows secondary (level 2) KEGG pathway classifications, and the y-axis indicates the number of metabolites potentially involved in each pathway. Colors correspond to primary (level 1) KEGG classifications.

**Figure 2:**
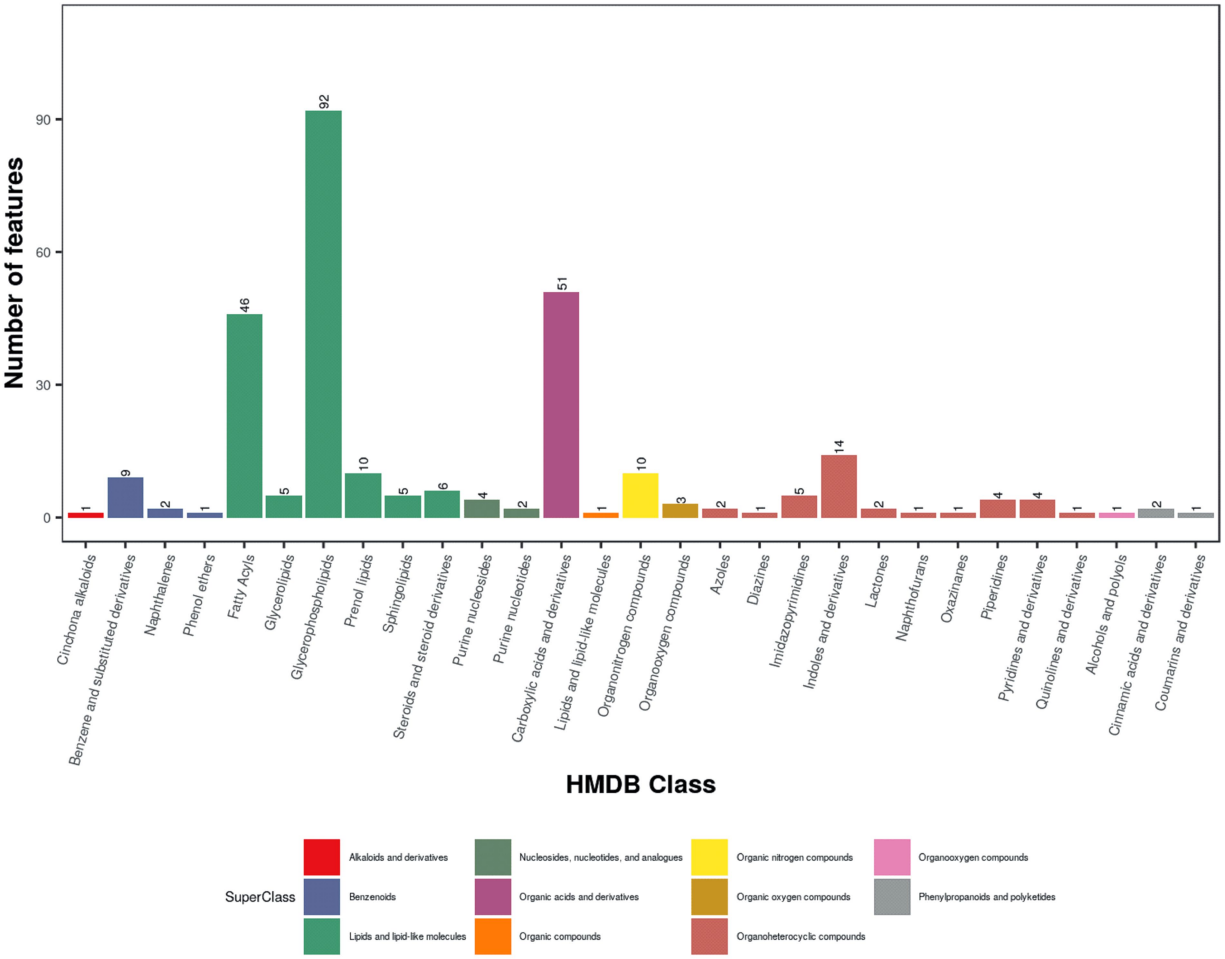
HMDB classification of MS2 metabolites in positive ion mode (POS). HMDB classification map of serum metabolites detected in fattening lambs under positive ion mode.

A total of 11,090 ions were detected in POS mode, with 4,715 annotated, and 7,207 in NEG mode, with 3,226 annotated (Table 1). Quality control was ensured using total ion chromatogram (TIC; Supplementary Figure S1), extracted ion chromatogram (EIC; Supplementary Figure S2), m/z-retention time distribution (Supplementary Figure S3), m/z difference range (Supplementary Figure S4), and retention time difference range (Supplementary Figure S5) for peak alignment. Metabolites were classified using the Human Metabolome Database (HMDB) and Kyoto Encyclopedia of Genes and Genomes (KEGG). In this study, 21,230 (POS) and 14,776 (NEG) metabolites were categorized into 24 superclasses in HMDB, with lipids and lipid-like molecules dominating (13,936 in POS, 9,482 in NEG; Figure 1A; Supplementary Figure S6A), followed by phenylpropanoids and polyketides, organoheterocyclic compounds, organic acids and derivatives, and benzenoids. For MS1 data, 3,121 (POS) and 2,060 (NEG) possible metabolites were assigned to 38 KEGG primary pathways, with metabolism-related pathways (78.7%) predominant, including global and overview maps (28.5%), lipid metabolism (12.6%), amino acid metabolism (10.8%), metabolism of cofactors and vitamins (7.4%), and carbohydrate metabolism (6.3%; Figure 1B; Supplementary Figure S6B; Table 1). Among secondary metabolites, approximately 60% (164/288) were lipids and lipid-like molecules, with glycerophospholipids (56.1%), fatty acyls (28.1%), and prenol lipids (6.1%) being the most abundant, followed by organic acids and derivatives (17.8%), organoheterocyclic compounds (12.2%), organic nitrogen compounds (3.5%), and benzenoids (4.2%; Figure 2).

**Table 1:** Total ion counts and identification statistics*.

Mode	All features	Annotated features	MS2 matches	MS1-HMDB matches	MS1-KEGG matches
POS	11,090	4,715	332	4,128	3,121
NEG	7,207	3,226	263	2,441	2,060
Total	18,297	7,941	595	6,569	5,181

*Mode refers to the ionization mode used in mass spectrometry: POS (positive ion mode) and NEG (negative ion mode). All Features indicates the total number of substances extracted using XCMS software. Annotated Features refers to the number of substances annotated based on primary and/or secondary mass spectrometry data. MS2 Matches denotes the number of substances matched by both the precursor ion (MS1) and the fragment ions (MS2). MS1-HMDB Matches indicates matches based on MS1 data with the Human Metabolome Database (HMDB). MS1-KEGG Matches indicates matches based on MS1 data with the KEGG database.

### 3.2 Variation in metabolite abundance associated with RPC

**Figure 3:**
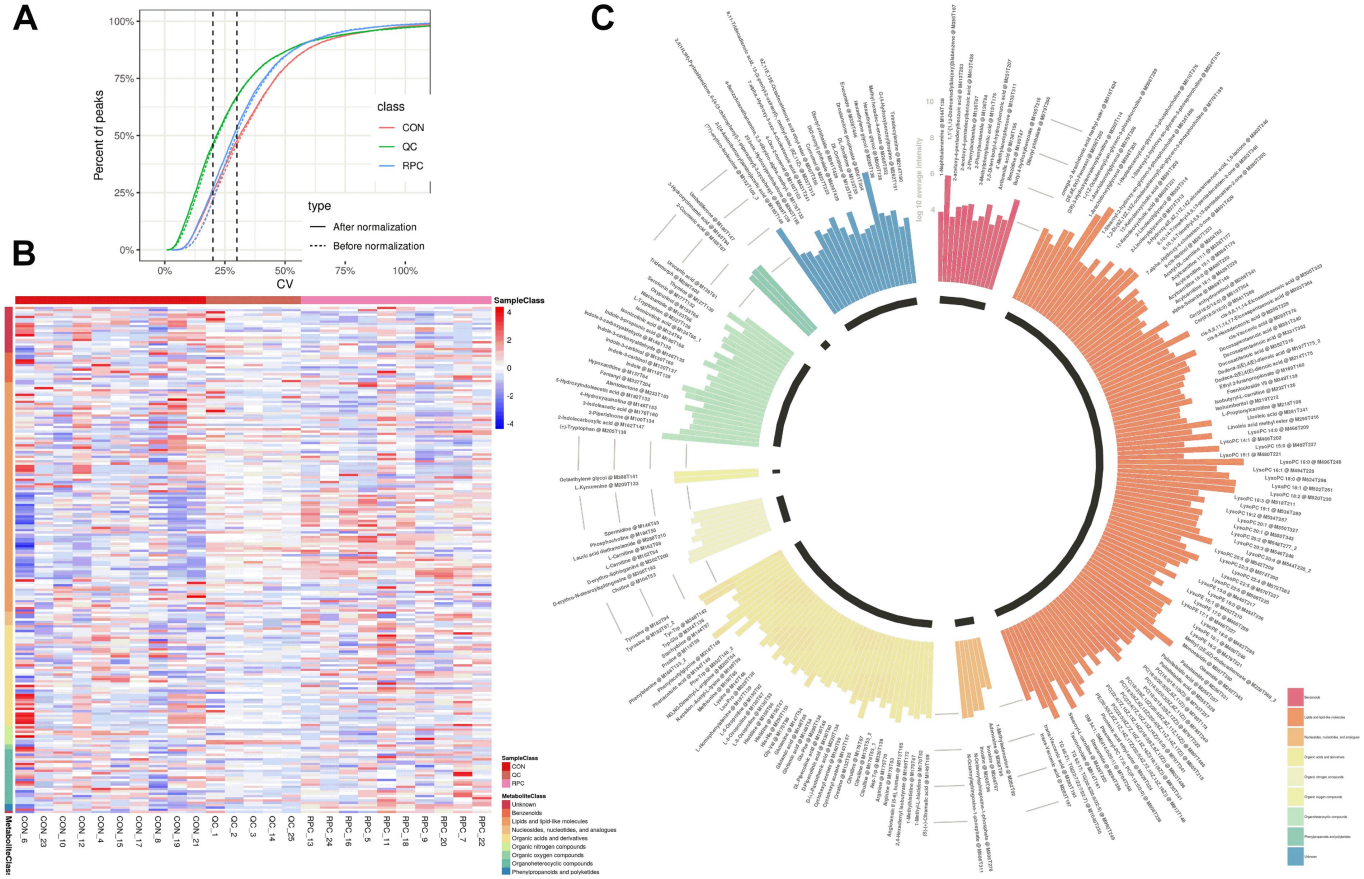
Abundance variation of rumen-protected choline (RPC) metabolites in fattening lambs. **(A)** Coefficient of variation (CV) analysis of metabolites in quality control (QC) samples after normalization. **(B)** Cluster heatmap of differential metabolites between treatment groups. Red denotes higher metabolite levels, and blue denotes lower levels. **(C)** Circular bar graph showing the average intensity of secondary metabolites. Colors indicate different metabolite classes.

Metabolic changes induced by RPC were evaluated using quality control parameters, including the coefficient of variation (CV) and mean metabolite intensity. All CV values were below 30%, indicating high analytical reproducibility (Figure 3A). Hierarchical cluster analysis of 220 DAMs revealed distinct patterns, with red indicating significantly increased metabolite levels and blue indicating decreased levels in the RPC group compared to the control (Figure 3B). Heatmaps confirmed robust clustering within groups, validating the reliability of the identified metabolites. The primary metabolite classes included benzenoids, lipids and lipid-like molecules, nucleosides, nucleotides and analogs, organic acids and derivatives, organic nitrogen compounds, organic oxygen compounds, organoheterocyclic compounds, phenylpropanoids and polyketides. The average intensity of high-confidence secondary metabolites showed a predominance of lipids and lipid-like molecules, organic acids and derivatives, and organic oxygen compounds, which are associated with fat metabolism, amino acid transport, and energy metabolism (Figure 3C).

### 3.3 Identification and comparison of differentially abundant metabolites

**Figure 4:**
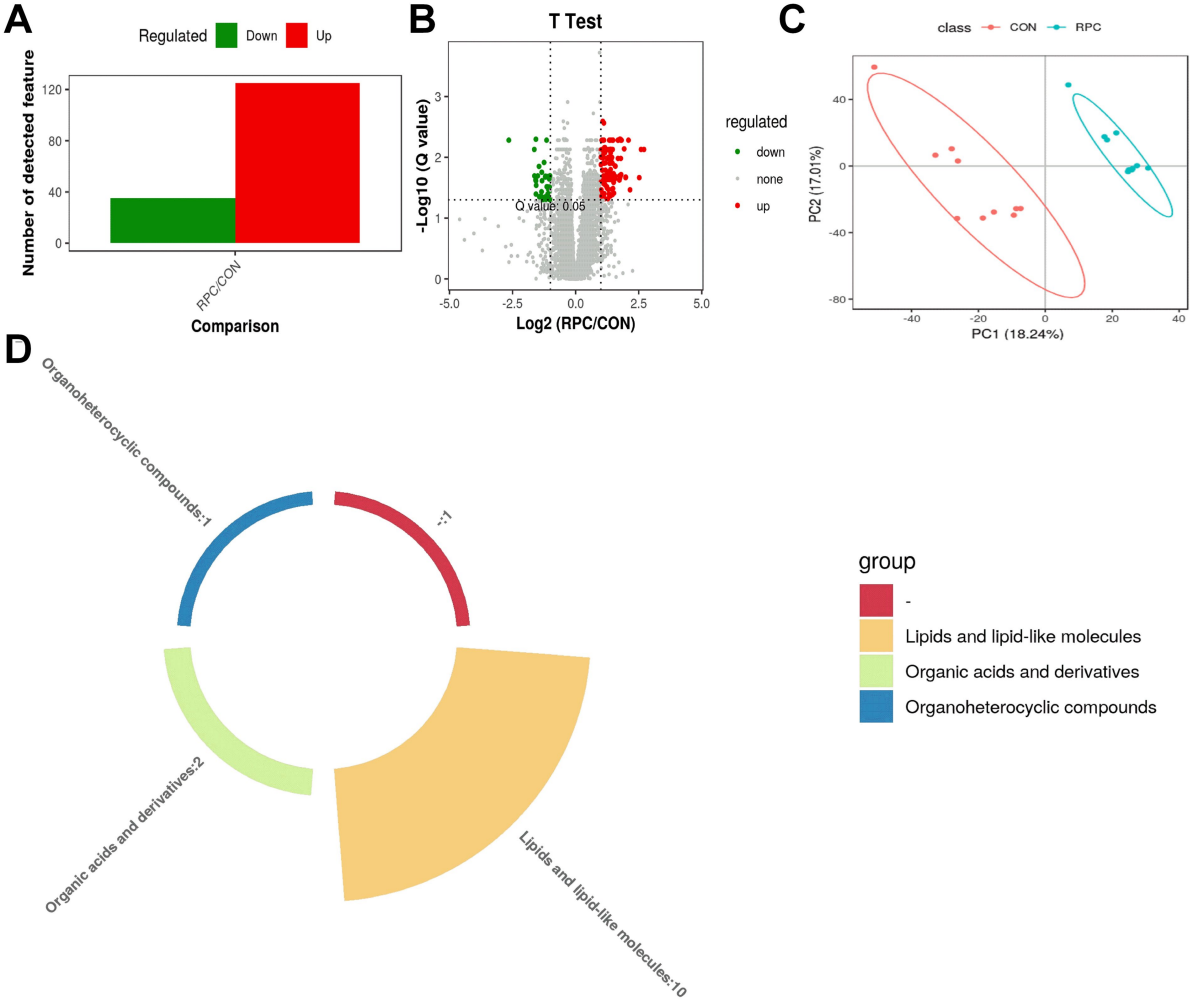
Identification and comparison of differentially abundant serum metabolites. **(A)** Statistical chart of differential metabolites. **(B)** Volcano plot of metabolite differences between RPC and control groups. The y-axis shows –log₁₀(*p*-value); the dashed line marks the significance threshold (*p* = 0.05, i.e., −log₁₀(0.05) ≈ 1.3). Green dots indicate significantly downregulated metabolites, red dots indicate upregulated ones, and gray dots represent non-significant differences. **(C)** Partial least squares discriminant analysis (PLS-DA) score plot of serum samples in positive ion mode. **(D)** Classification of differentially abundant serum metabolites between RPC and control lambs. Different colors represent distinct metabolite classes.

From 13,029 high-quality metabolites (Table 2), 300 were significantly altered (253 upregulated, 47 downregulated), with 160 annotated metabolites identified as DAMs (VIP > 1.0, *p* < 0.05, fold change > 2 or < 0.5), distinguishing the RPC group from the control group. Of these, 125 DAMs were up regulated (fold change > 2) and 35 were down regulated (fold change < 0.5; Figure 4A). A volcano plot illustrated significance, with metabolites above a -log_10_(*p*-value) threshold of 1.30 (equivalent to *p* < 0.05) considered significantly different (Figure 4B). Red dots represented up regulated metabolites, green dots down regulated, and gray dots non-significant changes. Partial least squares-discriminant analysis (PLS-DA) showed clear separation between the control and RPC groups without overlap, confirming significant differences in metabolic profiles (Figure 4C). A pie chart highlighted that lipids and lipid-like molecules were the most abundant DAMs, followed by organic acids and derivatives and organoheterocyclic compounds (Figure 4D).

**Table 2:** Feature detection and differential statistics*.

Mode	All features	High-quality features	Upregulated	Downregulated
POS	11,090	7,448	125	35
NEG	7,207	5,581	128	12
Total	18,297	13,029	253	47

*Mode refers to the detection mode of the mass spectrometer: POS (positive ion mode) and NEG (negative ion mode). All Features include all detected ions based on XCMS extraction. High-Quality Features are ions with fewer than 80% missing values in samples and fewer than 50% in quality control (QC) samples; only these were used for further statistical analysis. Upregulated and Downregulated represent the number of significantly altered features between the control (CON) and rumen-protected choline (RPC) supplementation groups.

**Figure 5:**
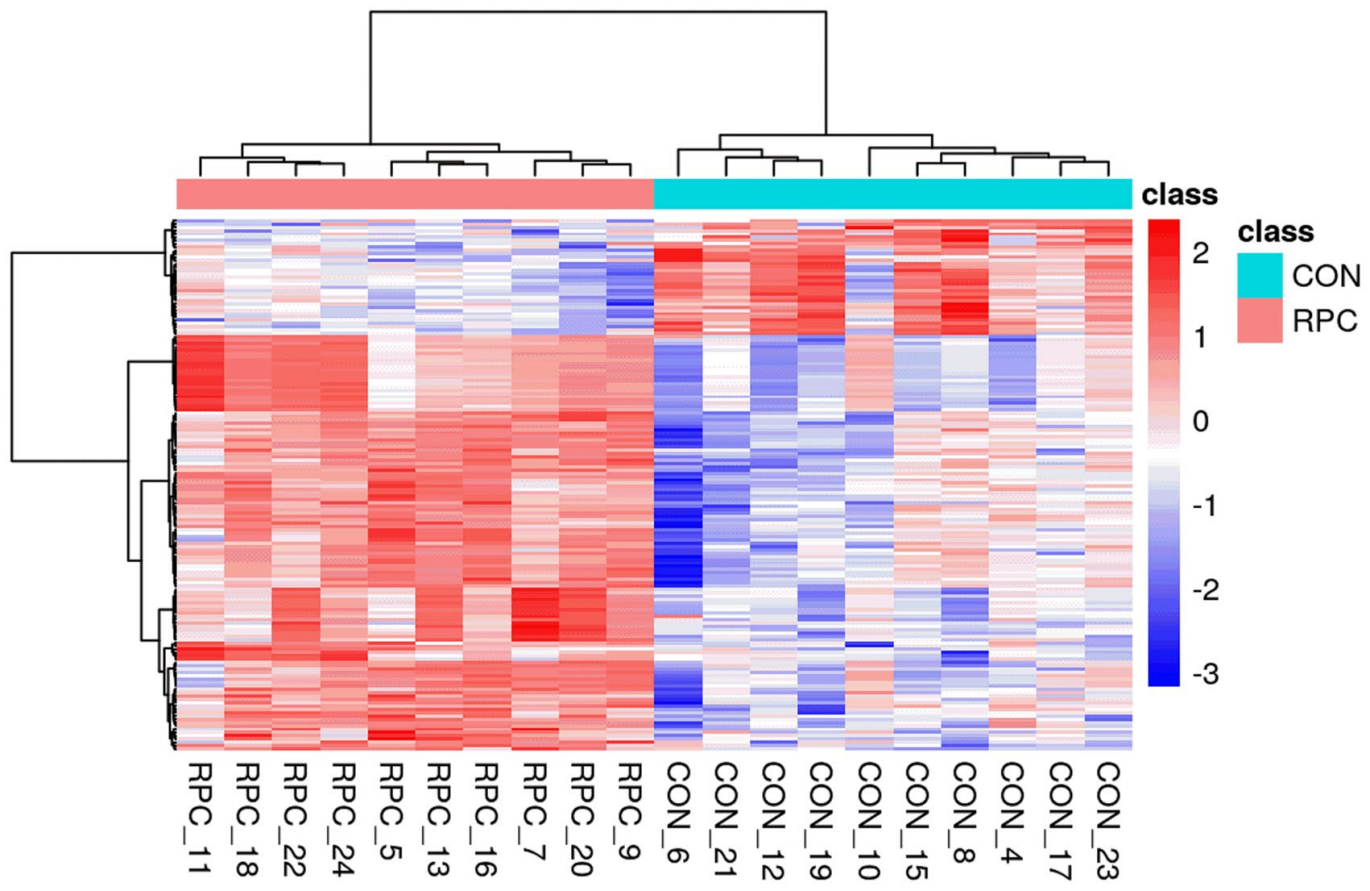
Heatmap of differential serum metabolites between RPC and control groups. RPC: rumen-protected choline group; CON, control group. The x-axis represents individual lambs, and the y-axis shows metabolite abundances. The color scale reflects relative abundance, with red indicating higher levels and blue indicating lower levels.

**Figure 6:**
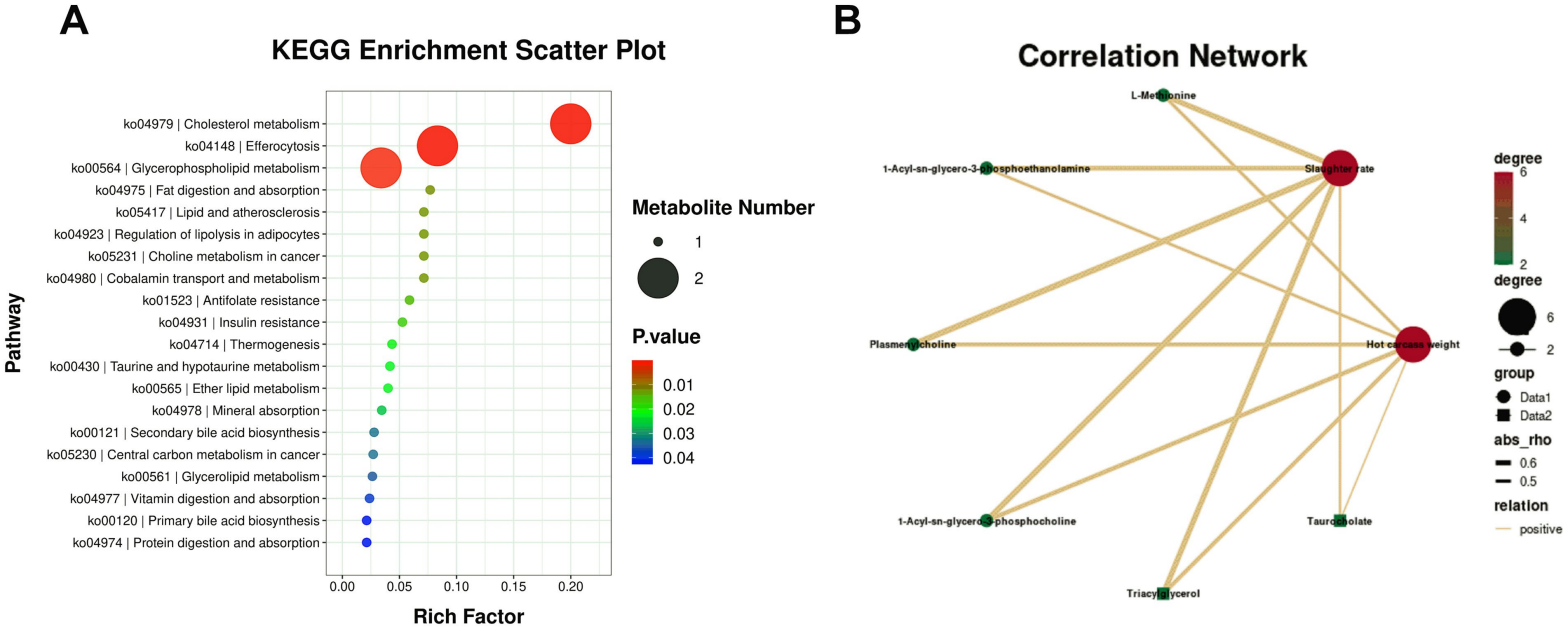
KEGG pathway enrichment and correlation analysis of differentially abundant serum metabolites (DAMs). **(A)** Bubble plot showing enriched KEGG pathways in the rumen-protected choline (RPC) group compared to the control group. Dot size represents the number of enriched metabolites, and the Rich Factor indicates the proportion of differentially abundant metabolites relative to all metabolites in each pathway. **(B)** Correlation network depicting relationships between differentially abundant metabolites (DAMs, green dots) and slaughter traits (red dots). Solid lines indicate positive correlations, and dashed lines indicate negative correlations, with values in the accompanying map representing correlation coefficients.

Hierarchical clustering of the 160 DAMs was visualized in a heatmap, with the control group (blue) and RPC group (red) showing distinct abundance patterns (Figure 5). KEGG enrichment analysis of these DAMs (*p* < 0.05) identified 33 enriched pathways, including cholesterol metabolism, glycerophospholipid metabolism, fat digestion and absorption, regulation of lipolysis in adipocytes, and taurine and hypotaurine metabolism (Figure 6A). Six key DAMs, triacylglycerol, L-methionine, plasmenylcholine, taurocholate, 1-acyl-sn-glycero-3-phosphoethanolamine, and 1-acyl-sn-glycero-3-phosphocholine, were identified as potential biomarkers for assessing RPC’s impact on growth and slaughter performance in fattening lambs. Based on the correlation coefficients between key metabolites and both HCW and slaughter rate, a correlation network diagram was constructed. The results indicated that all six key metabolites exhibited significant positive correlations with HCW (*p* < 0.05) and slaughter rate (*p* < 0.05; Figure 6B).

## 4 Discussion

Supplementation with RPC has been increasingly associated with improvements in growth performance, nutrient utilization, and carcass traits in ruminants. For instance, Habeeb (20) reported that RPC supplementation enhanced dry matter intake and feed conversion efficiency in goats. Similarly, RPC reduced the feed-to-gain ratio, improved nutrient digestibility, and increased average daily gain (ADG) and feed efficiency in sheep (13, 20, 24). These outcomes are consistent with the findings of Kawas et al. (24), who demonstrated improved feed utilization efficiency with RPC supplementation in feedlot lambs.

Our previous study (22) found that supplementing 5 g/kg DM RPC did not significantly improve growth performance or slaughter metrics but showed a trend toward increased HCW and slaughter rate, suggesting possible benefits in carcass yield. Other work in Hu sheep also noted improvements in meat quality and marbling scores following RPC administration, likely due to increased muscle density and HCW (25). As HCW is a critical index of commercial meat yield and correlates positively with subcutaneous fat thickness, backfat thickness, and longissimus muscle area (26), these findings support the hypothesis that RPC contributes to carcass quality. In our study, significantly higher abdominal fat content in the RPC group suggests a redistribution of fat stores, potentially favoring intramuscular fat (IMF) deposition over visceral fat such as perirenal fat, thereby enhancing meat quality.

Although no significant differences in ADG, dry matter intake, or slaughter traits were observed in our previous study (22), the present metabolomics analysis revealed substantial alterations in serum lipid, amino acid, and energy metabolic pathways. The upregulation of metabolites such as triacylglycerol and plasmenylcholine implies that RPC may influence systemic metabolic functions. Under optimal feeding and management conditions, these metabolic shifts could potentially enhance animal productivity and meat quality. These findings warrant further investigation.

To clarify the underlying biological mechanisms, untargeted liquid chromatography–tandem mass spectrometry (LC–MS/MS) was used to profile the serum metabolome. LC–MS/MS’s sensitivity enabled the identification of differentially abundant metabolites (DAMs), offering insight into RPC’s impact on nutrient metabolism.

### 4.1 Influence of RPC on amino acid biosynthesis, mineral absorption, and protein digestion

Neutral detergent fiber (NDF) digestibility is crucial for ruminant energy availability and rumen microbial activity (27). Our previous work (22) showed that RPC significantly improved NDF digestibility. Similarly, betaine, a key choline metabolite, has been shown to enhance fiber fermentation and mineral absorption (28) by modulating intracellular signaling pathways, including cytosolic calcium influx and ERK activation (29).

As a methyl donor, choline can partially substitute for methionine in transmethylation reactions, facilitating the remethylation of homocysteine to methionine via the betaine-homocysteine methyltransferase (BHMT) pathway (30). In the current study, increased L-methionine levels were detected in the mineral absorption pathway, supporting enhanced protein digestion. Choline also exhibits antioxidant effects and promotes tissue growth via its methyl group donation (31).

Moreover, RPC may modulate intestinal microbiota by increasing populations of lactic acid bacteria, thereby promoting short-chain fatty acid (SCFA) production and enhancing mineral bioavailability (32). However, excessive methionine intake can suppress beneficial microbes such as Roseburia and Blautia while favoring pro-inflammatory taxa, increasing hydrogen sulfide (H₂S) production via the transsulfurization pathway (33). Elevated H₂S may inhibit SCFA-producing bacteria and reduce acetate and butyrate levels (33), aligning with our prior post-feeding observation of dose-dependent SCFA reductions under RPC supplementation (22). This microbial imbalance may impair fiber digestion and exacerbate intestinal inflammation. Nonetheless, RPC may indirectly improve protein digestibility by promoting secretion of proteolytic enzymes like trypsin and chymotrypsin through microbiota-derived signals (34, 35).

### 4.2 Influence of RPC on glycerophospholipid and ether lipid metabolism

Choline is the precursor for phosphatidylcholine (PC) synthesis, central to glycerophospholipid metabolism. In our study, elevated levels of 1-acyl-PC suggest enhanced phospholipid synthesis via the CDP-choline pathway, potentially improving intestinal nutrient absorption and epithelial membrane integrity (36). In addition, phosphatidylethanolamine (PE), derived from 1-acyl-sn-glycero-3-phosphoethanolamine, may be synthesized through PLA2G-mediated pathways (37), and its accumulation indicates increased lecithin production under RPC (38).

However, excessive RPC (e.g., ≥0.75% of diet) may trigger endoplasmic reticulum stress and downregulate fatty acid oxidation genes like ACC, negatively affecting growth (36). RPC also altered muscle lipid composition, increasing unsaturated fatty acids (e.g., oleic and linoleic acid), which are known to reduce shear force and drip loss post-slaughter (36).

Our previous study (22) revealed inconsistencies across muscle types, with some muscles potentially showing increased IMF based on serum lipid metabolite profiles (e.g., triacylglycerol, plasmenylcholine), while others, such as certain hindquarter muscles, exhibited reduced fat content and 65% higher drip loss. These serum lipid metabolites, particularly phosphatidylcholine-related compounds, may influence flavor, as lipid profiles are known to contribute to meat flavor across muscle types (39). These discrepancies may stem from differences in muscle-specific lipid metabolism or from RPC dosage effects (5 g/kg DM). Future work should investigate optimal inclusion rates and tissue-specific responses to RPC. Supporting this, Çelik and Muruz (13) demonstrated that RPC at varying energy levels improved lamb marbling scores. Conversely, combining RPC with rumen-protected fat increased feed intake and altered fatty acid profiles without improving growth, indicating context-dependent responses.

### 4.3 Influence of RPC on fat digestion, glycerolipid metabolism, and lipolysis

In the present study, dietary supplementation with RPC, compared with the CON group, was found to modulate HCW and slaughter rate by altering metabolites associated with glycerophospholipid and glycerolipid metabolism, as well as other metabolic pathways. Triacylglycerol (TAG), the dominant form of stored fat in adipocytes, is central to fat digestion and glycerolipid metabolism (40, 41). In our study, RPC increased muscle and hepatic TAG accumulation, consistent with the findings of Elek et al. (42) and Liang et al. (43), who noted that excess TAG and diacylglycerol (DAG) may impair insulin sensitivity and muscle function.

Following RPC supplementation, plasma TAG and choline ion levels rose (44), supporting enhanced VLDL synthesis and hepatic lipid metabolism (45). Notably, lipid turnover in lactating dairy cows may differ from growing lambs, and choline’s impact on triglycerides appears dose-dependent (46).

Additionally, RPC promotes glycogen synthesis and inhibits gluconeogenesis via PI3K-AKT-GSK3 signaling, while upregulating GLUT4-mediated glucose uptake (47). These changes may increase free fatty acids (FFA) in muscle, which serve as energy substrates but may exacerbate intramyocellular lipid accumulation and associated dysfunction (43).

RPC also influences bile acid (BA) metabolism. Elevated taurocholate levels in our RPC group suggest enhanced BA secretion, facilitating TAG emulsification and absorption. This aligns with Sun et al. (48) and da Silva et al. (49), who showed increased bile acid levels (including cholic acid conjugates) under RPC supplementation and impaired lipid digestion in choline-deficient animals. The elevated taurine concentration observed in methionine-supplemented animals may also support BA synthesis (50).

BA metabolism is regulated by FXR, which inhibits adipogenesis via LXR/SREBP-1c and modulates glucose uptake via GLUT2 inhibition and GLP-1 stimulation (51, 52). The BA, methylcysteine complex, acting as an FXR antagonist, has also been reported to reduce hepatic lipid accumulation while promoting BA production (53).

## 5 Conclusion

This study demonstrates that RPC supplementation in fattening lambs modulates multiple metabolic pathways, including (1) enhanced protein utilization through increased methionine deposition and protein hydrolase secretion; (2) improved intestinal microbiota balance, promoting SCFA production and mineral absorption; (3) increased dietary fiber fermentation; and (4) regulation of glycerophospholipid and glycerolipid metabolism, which may lead to higher IMF content and improved meat quality. Untargeted LC–MS/MS metabolomics analysis identified 160 differentially abundant metabolites, with key biomarkers (e.g., triacylglycerol, L-methionine, plasmenylcholine) enriched in pathways related to lipid, amino acid, and energy metabolism. These results provide mechanistic insights into how RPC influences growth performance and carcass traits in ruminants. However, excessive methionine accumulation may impair SCFA production and dietary fiber utilization, potentially leading to intestinal inflammation. The influence of dietary calcium on intestinal microbiota distribution remains poorly understood and warrants further investigation. Future studies should focus on optimizing RPC dosage and clarifying its long-term effects on microbial and metabolic profiles to support its effective use in ruminant nutrition and meat quality enhancement.

## Data Availability

The metabolomic data generated in this study have been deposited in the OMIX, China National Center for Bioinformation/Beijing Institute of Genomics, Chinese Academy of Sciences (https://ngdc.cncb.ac.cn/omix/release/OMIX012211). (BioProject: PRJCA047602).

## Footnotes

^1^ https://www.hmdb.ca/

^2^ https://www.kegg.jp/

## Supplementary material

SUPPLEMENTARY FIGURE 1 Total Ion Chromatogram (TIC). **(A)** TIC in positive ion mode (POS). **(B)** TIC in negative ion mode (NEG). The chromatograms plot retention time on the x-axis and ion intensity on the y-axis, representing the summed signal of all detected ions at each time point. The TIC provides a macroscopic view of metabolite separation during liquid chromatography. Greater intensity overlap indicates higher instrument stability.

SUPPLEMENTARY FIGURE 2 Representative extracted ion chromatograms (EICs) of identified metabolites. **(A)** EIC from the control (CON) group. **(B)** EIC from the rumen-protected choline (RPC) group. The chromatograms were extracted from raw mass spectrometry data based on their identified metabolites. The x-axis indicates retention time, while the y-axis shows ion intensity. Peaks of different colors represent individual samples within each group.

SUPPLEMENTARY FIGURE 3 Mass-to-charge ratio (m/z) and retention time (RT) distribution plots. **(A)** Distribution in positive ion mode (POS). **(B)** Distribution in negative ion mode (NEG). In each plot, the x-axis represents the retention time, the y-axis represents the mass-to-charge ratio (m/z), and each point corresponds to a detected compound. The color gradient indicates the density of compounds within specific regions of the plot.

SUPPLEMENTARY FIGURE 4 Mass-to-charge ratio (m/z) alignment range of metabolites. **(A)** m/z alignment range in positive ion mode (POS). **(B)** m/z alignment range in negative ion mode (NEG). These diagrams illustrate the m/z range used for each metabolite during peak alignment.

SUPPLEMENTARY FIGURE 5 Retention time (RT) alignment range of metabolites. **(A)** RT alignment range in positive ion mode (POS). **(B)** RT alignment range in negative ion mode (NEG). These plots illustrate the retention time range used for peak alignment of each metabolite.

SUPPLEMENTARY FIGURE 6 HMDB superclass classification and KEGG pathway annotation of MS1 metabolites in negative ion mode (NEG). **(A)** HMDB Superclass Classification: Metabolites identified at level one were categorized into 24 HMDB superclasses. The x-axis indicates the superclasses, and the y-axis shows the number of identified metabolites within each category. **(B)** KEGG Pathway (Level 2): The x-axis represents the second-level KEGG pathway categories, while the y-axis indicates the number of metabolites potentially involved in each pathway. Colors correspond to the first-level KEGG pathway classifications.
